# Development of a range of fluorescent reagentless biosensors for ATP, based on malonyl-coenzyme A synthetase

**DOI:** 10.1371/journal.pone.0179547

**Published:** 2017-06-21

**Authors:** Renée Vancraenenbroeck, Simone Kunzelmann, Martin R. Webb

**Affiliations:** 1 The Francis Crick Institute, London, United Kingdom; 2 Structural Biology Science Technology Platform, The Francis Crick Institute, London, United Kingdom; University of Colorado Anschutz Medical Campus, UNITED STATES

## Abstract

The range of ATP concentrations that can be measured with a fluorescent reagentless biosensor for ATP has been increased by modulating its affinity for this analyte. The ATP biosensor is an adduct of two tetramethylrhodamines with MatB from *Rhodopseudomonas palustris*. Mutations were introduced into the binding site to modify ATP binding affinity, while aiming to maintain the concomitant fluorescence signal. Using this signal, the effect of mutations in different parts of the binding site was measured. This mutational analysis revealed three variants in particular, each with a single mutation in the phosphate-binding loop, which had potentially beneficial changes in ATP binding properties but preserving a fluorescence change of ~3-fold on ATP binding. Two variants (T167A and T303A) weakened the binding, changing the dissociation constant from the parent’s 6 μM to 123 μM and 42 μM, respectively. Kinetic measurements showed that the effect of these mutations on affinity was by an increase in dissociation rate constants. These variants widen the range of ATP concentration that can be measured readily by this biosensor to >100 μM. In contrast, a third variant, S170A, decreased the dissociation constant of ATP to 3.8 μM and has a fluorescence change of 4.2 on binding ATP. This variant has increased selectivity for ATP over ADP of >200-fold. This had advantages over the parent by increasing sensitivity as well as increasing selectivity during ATP measurements in which ADP is present.

## Introduction

Fluorescent reagentless biosensors are a class of probes for measuring concentrations of molecules: they are essentially single molecular species that couple detection of the analyte with a fluorescent signal. Here the biosensor is a covalent adduct of a binding protein and fluorophore [1]. Potential advantages of this type of probe are high sensitivity due to the fluorescence signal and rapid response, depending on the rate of binding the analyte. The fact that only a single molecular species is required means that the system under study is minimally modified unlike, for example, coupled enzyme assays in which the presence of several different additional species are required.

Recently, we described the development of a fluorescent reagentless biosensor for ATP, based on malonyl-coenzyme A synthetase from *Rhodopseudomonas palustris* (RpMatB) [2]. RpMatB catalyzes the conversion of malonate and coenzyme A to AMP, pyrophosphate and malonyl-coenzyme A. RpMatB binds ATP at the interface between two domains. In particular, the C-terminal “lid” closes down on the ATP binding site [3]. The design of the ATP biosensor made use of that conformational change together with the reversible stacked dimer formation between two tetramethylrhodamines, covalently bound to RpMatB via two, strategically introduced cysteine point mutations [2]. Such stacking leads to fluorescence quenching[4–6] and requires close interaction between the rhodamines [5, 6]. The stacking of the two rhodamines was possible in the apoprotein, but disrupted as the protein conformation changes on ATP binding.

In addition to two cysteine mutations, the biosensor had a C106A mutation to eliminate background labeling at that cysteine and a K488A mutation to block the adenylation half-reaction of ATP and malonate to malonyl-AMP and pyrophosphate [3]. The resulting protein adduct, termed Rho-MatB, had essentially no enzyme activity, but bound ATP with a *K*_d_ of 6 μM. This ATP biosensor couples ATP binding to a 3.7-fold increase in fluorescence intensity and measures ATP concentrations in the low micromolar range. Of importance for many potential uses is the fact that this biosensor is selective for ATP over ADP: often ATP is generated from ADP.

The ability to assay ATP using such a biosensor to some extent mirrors the use of those for ADP, such as for measuring kinase activity, or inorganic phosphate, such as for ATPases. Sensing ATP production and ATP as a signalling molecule [7, 8] are the main goals of the biosensor. It is also possible to use the biosensor for sensing ATP as a substrate, thereby measuring its depletion: sometimes this is preferable over product measurement, for example when the product is present at high concentration throughout, or if coupled reactions are being assayed.

Fluorescent biosensors are each likely to have an optimal range of analyte concentrations that can be measured. In the case of biosensors, such as here, where the analyte binds to a protein, the dissociation constant of the analyte is a major determinant of the optimal range: the degree of binding, and hence the signal, changes maximally from several-fold below that dissociation constant to several fold above. The measurable range of analyte can, in principle, be changed in several ways.

Firstly, in some cases, simply using a much higher concentration of biosensor may be possible, so that its concentration is well above the analyte dissociation constant and so binding is quantitative. The measurable analyte concentration is now limited by the biosensor concentration [9]. This is likely to have limited application, in part because of the large amount of biosensor required, limiting practicality and potential introducing both solution and optical complications.

Secondly, the binding site of the biosensor can be modified by mutation, making use of structural information [10]. However, there is a challenge in this approach to maintain the fluorescence signal after mutation. In the case of MatB, the signal is likely to be directly dependent on the ATP-induced conformation change, so the latter must be maintained. This is the predominant approach used in this work. The most simple reason for changing the concentration range in this way, is to measure high concentrations without resorting to high biosensor usage. With small, sub-stoichiometric concentration of biosensor, the degree of saturation by analyte binding, and hence fluorescence, reflects its concentration. An example of a type of assay where this might be useful is measuring ATP usage by an enzyme with high K_m_-value for ATP. Furthermore a biosensor, used in this way, will only change the concentration of free ATP slightly.

It may also be possible to attenuate binding of the analyte through use of another ligand. This could be one that binds to another site on the protein, but affecting affinity at the analyte binding site. Alternatively a ligand that also binds in the analyte binding site, but without inducing the fluorescence change, will compete with the analyte and reduce the apparent affinity of the biosensor for the analyte. Indeed, this approach was inherent in a modular-designed biosensor [11]. We will briefly describe how AMP might be used in this way as it binds, presumably without inducing the lid-closing conformation change that is required for the fluorescence change.

The method chosen to change affinity was to mutate amino acids that were identified from the crystal structure as interacting with ATP [3]. Mostly these were predicted as unlikely to affect the lid closure: that is they are not positioned at the hinge or involved in interactions across the cleft, nor might they affect the rhodamines directly. Essentially, there was a survey of the effect of changing the active-site amino acids on ATP affinity, which was readily measured using the rhodamine fluorescence change. This survey revealed three new variants, one tighter and two weaker in ATP binding, resulting in the ability to measure ATP from sub-micromolar to >100 μM. If the affinity is decreased to allow measurements of higher ATP concentrations, a low, sub-stoichiometric concentration of biosensor can be used, minimizing biosensor usage [12]. Alternatively, increasing the affinity of Rho-MatB for ATP may allow measurements of lower ATP concentrations, thereby increasing sensitivity over the parent biosensor.

## Materials and methods

### Materials

Purine nucleotides were obtained from Sigma-Aldrich, except 99%-pure ADP was from Alfa Aesar. 5-iodoacetamidotetramethylrhodamine (5-IATR) [13] was a gift from J. Corrie (NIMR, London) or obtained from Anaspec (CA).

### Plasmids

Plasmid pRhoRpMatB (plasmid pTEV5 containing the coding sequence of RpMatB (Genbank accession number CAE25665.1) with the point mutations C106A, R286C, Q457C, K488A and an N-terminal His_6_-tag) was as described and now termed pTEV5_RpMatB_1 [2]. The QuikChange site-directed mutagenesis protocol (Stratagene) was used for single-site mutations. Plasmids were sequenced (GATC Biotech) to confirm the presence of the mutations. The three variant plasmids, containing additional S170A, T167A or T303A mutations, are termed pTEV5_RpMatB_2, pTEV5_RpMatB_3, and pTEV5_RpMatB_4 respectively. All four plasmids are available from Addgene.

### Preparation of RpMatB variants, labeled with tetramethylrhodamine

Protein expression in *Escherichia coli*, purification and labeling with tetramethylrhodamine were as described [2]. RpMatB variants were expressed in E. coli OverExpress C41(DE3) cells (Lucigen). The cell pellet of a 5 ml overnight culture, grown at 37°C and supplemented with 100 μg ml-1 ampicillin, was resuspended in 0.5 l of lysogeny broth, supplemented with ampicillin, in 5 l shaker flasks and cultured via vigorous shaking at 30°C to an optical density at 600 nm of 0.6–0.8 cm-1. Then 0.5 mM isopropyl-β-D-thiogalactoside was added to start induction at 30°C for 16 h. After induction, the cultures were cooled to 4°C and centrifuged at 3500 rpm for 30 min at 4°C (rotor JS 4.2, Beckman). The cell pellet was washed with 30 ml of ice-cold buffer (10 mM Tris.HCl pH 7.5, 300 mM NaCl), centrifuged at 3500 rpm for 30 min at 4°C, the supernatant discarded and the pellet stored at -80°C. Typically ~3 g wet weight of E. coli cells was harvested from 0.5 l culture.

The cell pellet was resuspended in 35 ml 30 mM Tris.HCl, 300 mM NaCl, 10 mM imidazole, 3 mM tris(2-carboxyethyl)phosphine, 2 mM phenylmethanesulfonyl fluoride, pH 8.0 and sonicated on ice using an ultrasonicator (VC505, Sonics) at 200 W for 5 times 30 s with a 5 s on / 5 s off pulse. The soluble fraction was collected by centrifugation at 35000 rpm for 45 min at 4°C (rotor 45 Ti, Beckman). The His6-tagged protein was purified at 4°C by immobilized metal ion affinity chromatography (1 ml HisTrap HP column, GE Healthcare) using an Äkta system (GE Healthcare). The resin was equilibrated with Buffer A (30 mM Tris.HCl, 300 mM NaCl, 10 mM imidazole, 1 mM tris(2-carboxyethyl)phosphine, pH 8.0). The sample was filtered (0.45 μm Minisart NML filter, Sartorius) and loaded onto the column at 0.5 ml min-1. The column was washed with 20 ml Buffer A and additionally with 20 ml of 95% Buffer A and 5% Buffer B (30 mM Tris.HCl, 300 mM NaCl, 250 mM imidazole, 1 mM tris(2-carboxyethyl)phosphine, pH 8.0) at a flow rate of 1 ml min-1. The protein was eluted with 20 ml of Buffer B at a flow rate of 1 ml min-1. Protein fractions were pooled (~2–4 ml) and further purified via size exclusion chromatography at 4°C using the HiLoad 16/60 Superdex 200 prep grade column (GE Healthcare) equilibrated with 30 mM Tris.HCl, 100 mM NaCl, 0.5 mM ethylenediaminetetraacetic acid, 5 mM dithiothreitol, 1 mM NaN3 at 1 ml min-1.

Fractions containing the protein were pooled and concentrated (VivaSpin 20 MWCO 10 kDa cut off, GE Healthcare) to ~10 mg ml-1. The protein concentration was determined from the absorbance at 280 nm using the extinction coefficient at 280 nm of 46300 M-1 cm-1 calculated from the sequence via Expasy Protparam2. The protein was drop-frozen in liquid nitrogen and stored at -80°C. Typically, 45 mg of protein was obtained from 3 g wet weight of cells.

Dithiothreitol was removed from ~40 mg of RpMatB using a PD10 desalting column (GE Healthcare) pre-equilibrated with Buffer L (30 mM Tris.HCl pH 7.5, 100 mM NaCl) at 20°C. 50 µM protein was incubated at 20°C with 225 µM 5-IATR (AnaSpec) in Buffer L using an end-over-end mixer for 90 min. Then 2 mM sodium-2-mercaptoethanesulfonate was added and incubation continued for 15 min. After centrifugation at 3500 rpm for 15 min at 4°C (Heraeus Biofuge), the supernatant was filtered through a 0.2 µm syringe filter (Acrodisc Syringe Filter with HT Tuffryn Membrane, Pall Life Sciences) and loaded onto a PD10 desalting column, equilibrated with Buffer Q1 (30 mM Tris.HCl pH 8.0, 25 mM NaCl) at ~20°C to remove free label.

The final biosensor was further purified via ion exchange chromatography at 4°C using a 1 ml HiTrap Q HP column (GE Healthcare), equilibrated in Buffer Q1 at 1 ml min-1. After sample loading, the column was washed with 90 ml Buffer Q1. The protein was eluted using a gradient from 100% Buffer Q1 to 50% Buffer Q1 and 50% Buffer Q2 (30 mM Tris.HCl pH 8.0, 1 M NaCl) over 25 ml followed by a gradient from 50% Buffer Q1 and 50% Buffer Q2 to 100% Buffer Q2 over 10 ml. Rho-MatB eluted as two overlapping peaks with identical mass and fluorescence properties. Both peaks were pooled and concentrated to ~5 mg ml-1 using a concentrator (Amicon Ultra-4 10 kDa cut off, Millipore).

While screening for a fluorescence change upon ATP binding, labeling was performed on a scale of ~2 mg of protein. RpMatB variants (100 µM) were incubated at 20°C with 2-fold excess MDCC over RpMatB for 35 min, or with 2-fold excess IDCC for 120 min, or with 4-fold excess 5-IATR or 6-IATR for 90 min. During screening, the labeled RpMatB variants were purified only using a PD10 desalting column.

The labeled protein concentrations were determined using the extinction coefficients for RpMatB (ε_280_ 46300 M^-1^ cm^-1^) and tetramethylrhodamine (ε_280_ 31000 M^-1^ cm^-1^ and ε_528_ 52000 M^-1^ cm-^1^) [13]. The protein was drop-frozen in liquid nitrogen and stored at -80°C. Labeling yields were up to 35%.

The concentrations of labeled RpMatB variants were determined using the extinction coefficient of the protein at 280 nm, calculated from the sequence via Expasy Protparam [14] and the extinction coefficients of tetramethylrhodamine: ε_280nm_ (31000 M^-1^ cm^-1^) and ε_528nm_ (52000 M^-1^ cm^-1^) [13]. Double labeling was confirmed by mass spectrometry and essentially no unlabeled protein was observed. Based on this and absorbance spectra, the double labeling is likely to be >90%

### Absorbance and fluorescence measurements

Absorbance was measured on a Jasco V-550 UV-Vis Spectrophotometer with a 1-cm pathlength cell. Fluorescent measurements were obtained on a Cary Eclipse spectrofluorometer (Agilent Technologies), using a 3-mm pathlength quartz cuvette (Hellma), unless otherwise mentioned. Excitation and emission slits were set at 5 nm. Protein and nucleotide concentrations and buffer conditions are given in the figure legends.

Data from titrations to measure nucleotide binding were corrected for dilution and analyzed with a quadratic binding curve using Grafit software [15]:

$$F=F_{min}+(F_{max}-F_{min})\times \frac{\left(K_{d}+L+P-\sqrt{{\left(K_{d}+L+P\right)}^{2}-4\times P\times L}\right)}{2\times P}$$

where *P* and *L* are the total concentrations of protein and ligand, respectively, *K*_d_ is the dissociation constant, and *F*_min_ and *F*_max_ are the fluorescence intensities of the free and ligand- bound protein, respectively.

### Stopped-flow measurements

These were carried out using a HiTech SF-61DX2 apparatus (TgK Scientific, Bradford-upon-Avon, UK) with a xenon-mercury lamp and operated by Kinetic Studio software (TgK Scientific). The excitation wavelength was 545 nm and there was an OG570 cut-off filter (Schott Glass) on the emission. The concentrations in the text and figures are those in the mixing chamber. Data were fitted to theoretical equations (single or double exponentials) using the Kinetic Studio software.

### Steady-state analysis of ATP production by pyruvate kinase

Steady-state activity measurements of pyruvate kinase (rabbit muscle from Sigma) were obtained using fluorescence on a CLARIOstar microplate reader (BMG Labtech). Assays were set up in 100 μl or 40 μl using 96-well using 384-well polystyrene microplates (low-protein-binding, Corning, USA) respectively.

### Sequence alignment

Structurally characterized ANL superfamily proteins (updated from [16]) were aligned based on their Uniprot entries [17], using the multiple sequence alignment program PROMALS3D [18].

## Results and discussion

### Identification of amino acid residues affecting binding of MgATP

**Fig 1:**
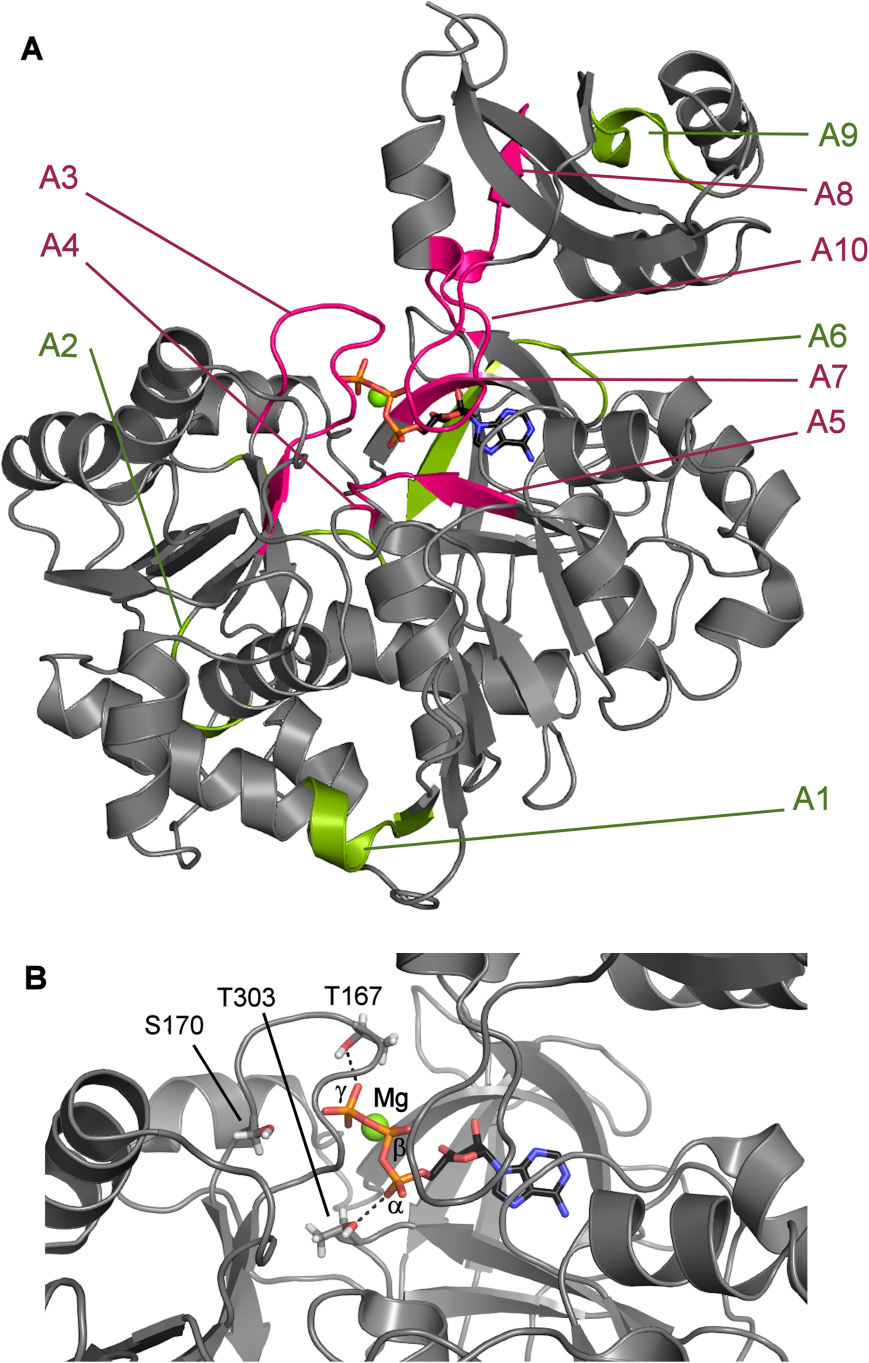
Crystal structure of RpMatB in the MgATP bound conformation, showing conserved motifs and position of binding site mutations. (A) Protein structure showing the conserved core motifs and MgATP [3]. The highly conserved core motifs (see also S1 Fig) are shown: structural motifs are coloured green, binding site motifs are pink. (B) ATP binding site, showing the position of the three mutations. The Mg, α-, β- and γ-Ps of ATP are labeled.

Those amino acid residues, which are important for the structural integrity and function of RpMatB, were considered for mutation. Our first goal was the RpMatB ATP binding site. Residues were chosen that were within 0.6 nm of MgATP as determined using the crystal structure of RpMatB in the MgATP-bound conformation [3]. In addition, sequence homology amongst the ANL family was considered (S1 Fig), so that mutations were avoided at highly conserved amino acids. Some of these residues belong to the A3, A4, A5, A7, A8 and A10 core motifs, which are known to play an important role in adenylate-forming enzymes [19]. The positions of these motifs are shown in Fig 1 and details of the motifs are in S1 Fig. The resulting positions that were subsequently mutated successfully are shown in Table 1 with their motifs and their location relative to the ATP structure.

**Table 1:** Fluorescence change and affinity for ATP binding to variants of Rho-MatB.

Variant	Motif	Position near ATP	*F*_+_/*F*_-_	*K*_d_ for ATP (μM)
Parent			2.92 ± 0.03	8.8 ± 0.3
T163A	A3	γ-P	1.81 ± 0.03	297 ± 27
S164A	A3	α-P	2.69 ± 0.02	17.4 ± 0.4
T166A	A3	β-P	2.04 ± 0.02	156 ± 7
T167A	A3	γ-P	2.87 ± 0.04	149 ± 7
T167S	A3	γ-P	3.25 ± 0.03	52 ± 1
R169A	A3	γ-P	2.75 ± 0.03	33 ± 1
S170A	A3	γ-P	3.85 ± 0.04	4.0 ± 0.1
H207A	A4	α-P	1.44 ± 0.01	224 ± 18
T208A		α-P	1.65 ± 0.02	52 ± 5
S277A		adenine	0.9 ^a^	—
E298A		adenine	1.1 ^a^	—
R299A		adenine	1.65 ± 0.01	2.4 ± 0.1
Y300A	A5	adenine	2.05 ± 0.02	11.1 ± 0.5
G301A	A5	adenine	0.8 ^a^	—
M302A	A5	ribose	1.47 ± 0.02	50 ± 5
T303A	A5	α-P	2.60 ± 0.02	42 ± 1
T303S	A5	α-P	1.73 ± 0.01	56 ± 3
I394A		adenine	1.1 ^a^	—
Q490A	A10	β-P, γ-P	2.78 ± 0.04	66 ± 3

**Fig 2:**
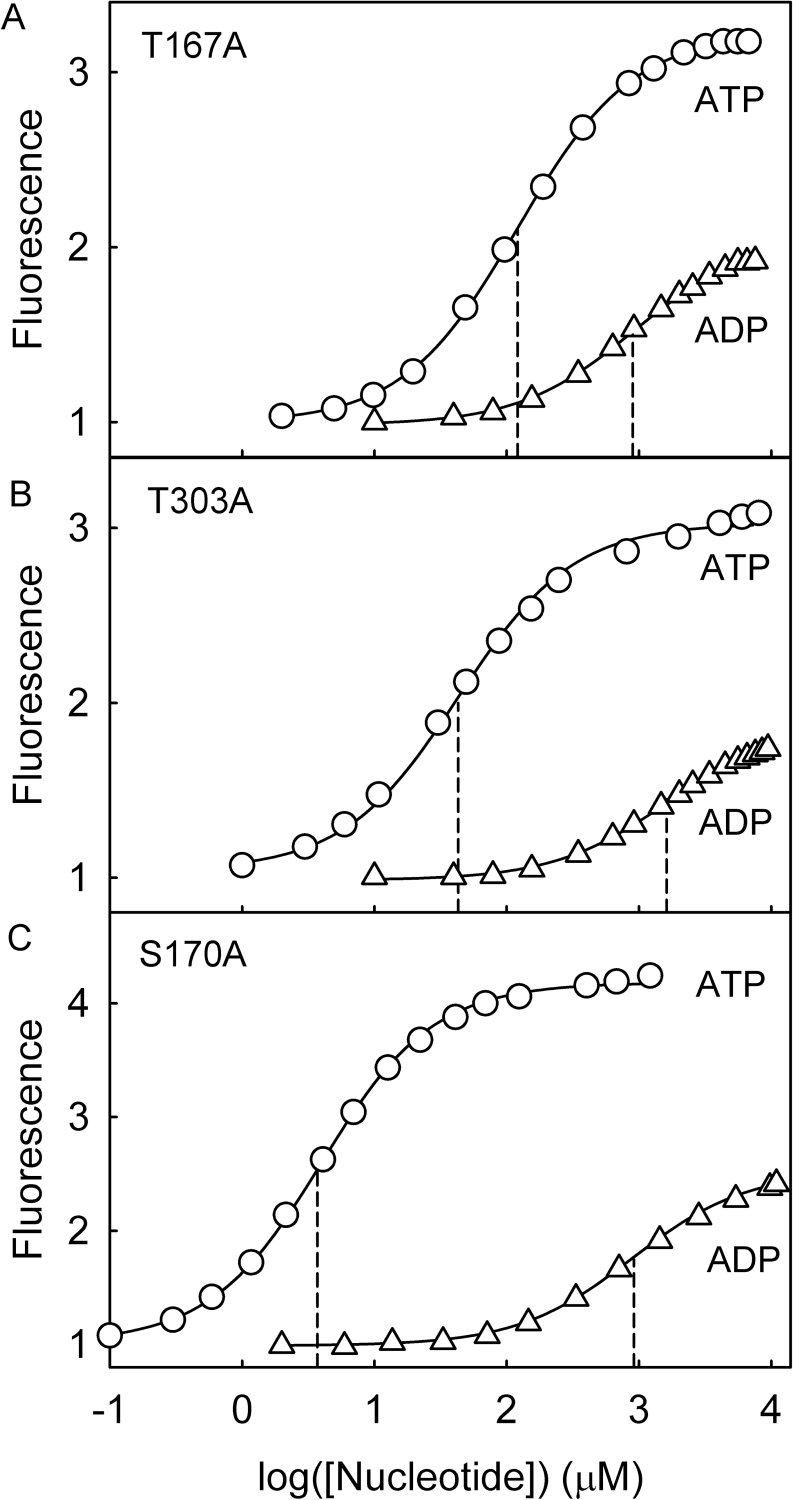
Nucleotide affinity for variants of Rho-MatB. Titration of ATP (circles) and ADP (triangles) to (A) 0.5 µM Rho-MatB T167A, (B) 0.5 μM Rho-MatB T303A; (C) 0.5 μM Rho-MatB S170A. Measurements are shown for one representative experiment and were in 50 mM Hepes pH 7.0, 100 mM NaCl, 10 mM MgCl_2_, 0.3 mg ml^-1^ bovine serum albumin at 20°C. Excitation was at 553 nm, emission at 571 nm. The dissociation constants and fluorescence ratios were obtained using a quadratic binding equation (see Methods) and are listed in Table 2. The drop lines are to indicate the dissociation constants. Data are normalized to the intensity in the absence of nucleotide.

Data are from a survey in 50 mM Tris.HCl pH 7.5, 150 mM NaCl, 10 mM MgCl_2_, 0.3 mg ml^-1^ bovine serum albumin at 20°C but without complete optimization of labeling and purification. The motifs are shown in Fig 1. The ratio of fluorescence at saturating ligand to that in the absence of ligand (*F*_+_/*F*_-_) and the ATP dissociation constant (*K*_d_) were determined by titrations as in Fig 2: the values and errors for both parameters come from a single screening titration, except as noted The parent Rho-MatB is the protein without any binding site mutations.

^a^ These ratios are from a single measurement at 750 μM ATP. As the fluorescence change was very low, no titration was performed.

### Preparation and testing of variants of Rho-MatB

To examine the effects of substituting these residues on Rho-MatB’s function, the individual residues were mutated to alanine on a background of (His_6_/C106A/R286C/Q457C/K488A)RpMatB [2]. In addition, more conservative threonine to serine substitutions were introduced at two positions (T167S and T303S), so potentially still allowing the new residues to interact with the triphosphate of ATP. The mutants were expressed and purified as for the parent RpMatB, but only variants that gave reasonable expression were purified and then labeled with 5-iodoacetamidetetramethylrhodamine (5-IATR).

The labeled proteins were then tested for a fluorescence change upon ATP binding in the presence of Mg^2+^ (Table 1). For variants showing a significant fluorescence change, the affinity for ATP was determined by titration (Table 1).

None of the variants of Rho-MatB responded to GTP under the same conditions (data not shown). Table 1 shows that the mutations, which least perturbed the fluorescence change on ATP binding, were all located near the triphosphate, particularly in the phosphate loop A3, and some of these caused the greatest decrease in affinity (T163A, T166A, T167A and H207A). This suggested that changes to this loop decreased binding but still allowed the full conformation change, which controls the interaction between rhodamines and therefore the fluorescence change. In contrast, all the mutations that cause almost complete loss of the fluorescence change were close to the adenosine.

Three variants that retained large changes in fluorescence signals but had potentially useful changes in affinity, relative to the parent were characterized for use as biosensors. S170A had a small but significant increase in affinity, so could be more sensitive at low concentrations. T167A and T303A, showed a large fluorescent increase upon binding MgATP, but also showed a significant reduction in ATP affinity, relative to the parent. In addition they gave good expression and labeling yields compared to the other mutants (data not shown). These three variants were chosen for further purification and their binding parameters were measured.

### Weak binding variants, T167A and T303A

**Fig 3:**
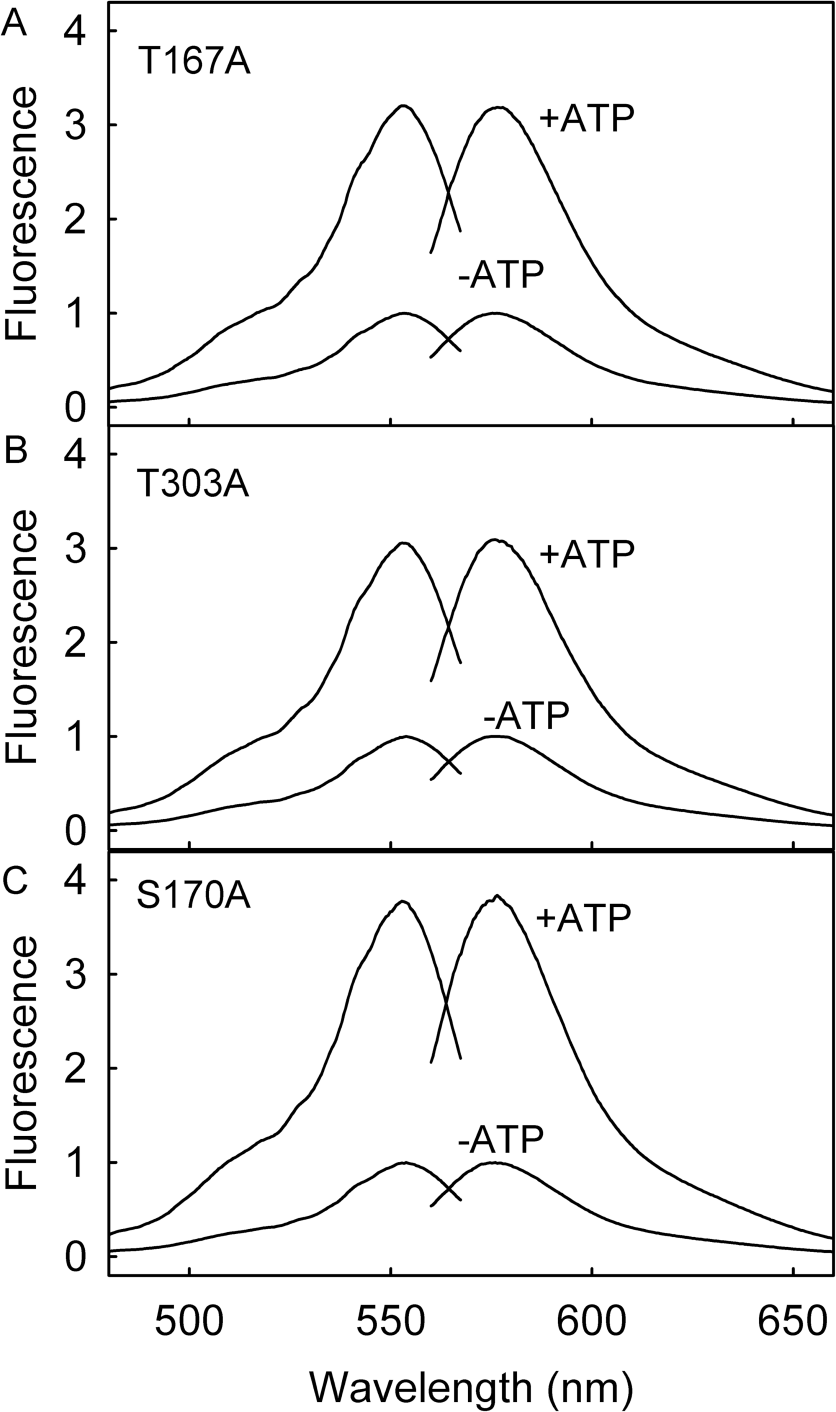
Fluorescence excitation and emission spectra of variants of Rho-MatB. (A) 1 µM Rho-MatB T167A with and without 5 mM ATP; (B) 1 μM Rho-MatB T303A with and without 3 mM ATP; (C) 1 μM Rho-MatB S170A with and without 0.5 mM ATP. These ATP concentrations were saturating for the variant. Solutions were in 50 mM Hepes pH 7.0, 100 mM NaCl, 10 mM MgCl_2_, 0.3 mg ml^-1^ bovine serum albumin at 20°C. Spectra are normalized to the maximum intensity in the absence of ATP.

The side chains of these amino acids are involved in hydrogen bonding between RpMatB and the triphosphate of ATP (Fig 1B). The change in fluorescence emission spectra on binding ATP are in Fig 3A and 3B. The absorbance spectra (S2A and S2B Fig) show changes on ATP binding typical for tetramethylrhodamine switching between mainly stacked to unstacked configuration, whereby there is a large change in ratio between the intensities of the two peaks [20, 21]. This behaviour was seen previously for the parent Rho-MatB and other proteins labeled with two rhodamines [2, 22–25].

The affinity for ATP and ADP was determined by measuring the fluorescence at different concentrations of the nucleotide (Fig 2A and 2B). Both variants show similar fluorescent changes with ATP compared to the parent Rho-MatB, but the dissociation constants (*K*_d_) are much higher (Table 2). In contrast, the affinity of ADP was much less affected by the mutations, remaining very weak (Table 2). The change in ATP affinity presumably reflects the importance of hydrogen-bonding interactions with the triphosphate, decreased by mutating to alanine.

**Table 2:** Fluorescence changes, dissociation constants and rate constants for ATP binding to variants of Rho-MatB.

Variant	*F*_+_/*F*_-_	*K*_d_ (ATP) (µM)	*k*_+2_ (µM^-1^ s^-1^)	*k*_-2_ (s^-1^)	*k*_+1_ (s^-1^)	*K*_d_ (ADP) (µM)	*K*_d_ ratio
Parent	3.9 ± 0.2	6.4 ± 0.6	1.8 ± 0.02	8.2 ± 0.2	0.88 ± 0.13	428 ± 50	69
T167A	3.2 ± 0.1	123 ± 4	1.83 ± 0.05	87 ± 4	0.56 ± 0.01	900 ± 28	7.3
T303A	2.7 ± 0.1	42 ± 2	1.60 ± 0.06	16 ± 4	0.60 ± 0.02	1835 ± 114	44
S170A	4.2 ± 0.1	3.8 ± 0.1	1.83 ± 0.10	9 ± 6	0.64 ± 0.02	887 ± 25	233

**Fig 4:**
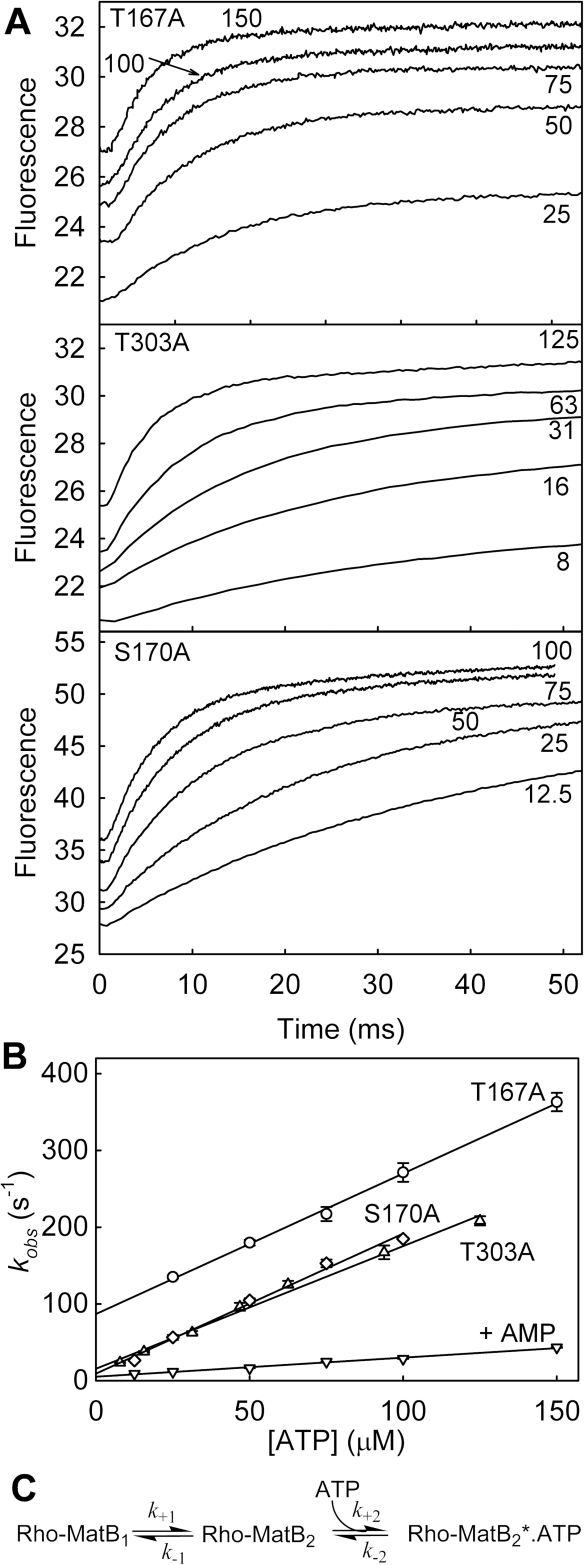
Association kinetics of ATP binding to Rho-MatB variants. Fluorescence time courses were measured by rapidly mixing 0.25 µM Rho-MatB with different concentrations of ATP in large excess (micromolar concentrations shown). Measurements for one representative dataset are shown and were obtained in 50 mM Hepes pH 7.0, 100 mM NaCl, 10 mM MgCl_2_, 0.3 mg ml^-1^ bovine serum albumin at 20°C. Time courses were obtained for two different time scales to show fast and slow phases. Slow phases are shown in S3 Fig. Note that the dead time of the stopped-flow instrument (the time taken for the mixed solution to reach the fluorescence observation chamber) is ~2 ms, so that the traces of the fast phase only show changes from that time. (A) Example traces for Rho-MatB T167A, T303A and S170A variants. (B) The average, observed rate constants are shown with standard errors. The fast phases were fit to single exponentials to give rate constants (*k*_obs_), increasing linearly with ATP concentration. “+AMP” is a set of measurements with the parent Rho-MatB in the presence of 500 µM AMP. (C) Conformational selection model for binding derived for Rho-MatB: step 1 is a conformation change of the apoprotein, this is followed by ATP binding. The star indicates the high fluorescence state. Pseudo-first order conditions used for the kinetic measurements give *k*_obs_ = *k*_+2_[ATP] + *k*_-2_: the slope (second order rate constant for association, *k*_+2_) and intercept (dissociation rate constant, *k*_-2_) are shown in Table 2.

Both the ratio of fluorescence at saturating ligand to that in the absence of ligand (*F*_+_/*F*_−_) and the dissociation constants (*K*_d_) were obtained from the data in Fig 2. Association kinetics were from Fig 4 and S3 Fig: *k*_+2_ is the second order association rate constant, *k*_-2_ is the dissociation rate constant and *k*_+1_ is the value obtained for the conformation change. The *K*_d_ ratio is for ADP/ATP. Values for the parent biosensor were taken at 25°C [2]. Note that these data were obtained with proteins that have been purified further than those used in Table 1 and are the fitted values from at least two replicates combined

### The effect of ADP on ATP response

**Fig 5:**
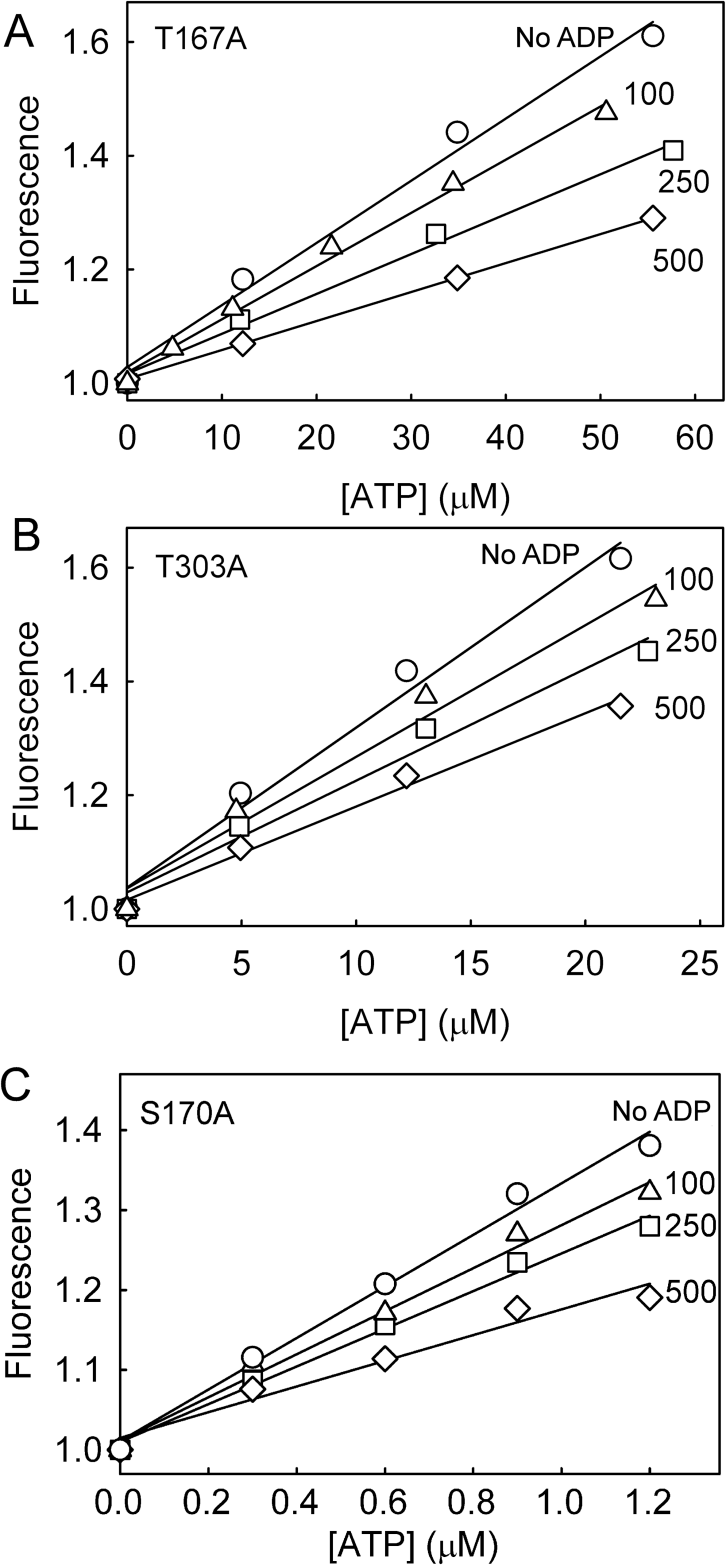
The effect of ADP on ATP-induced fluorescence. How ADP affects the ATP response was determined by measuring the fluorescence as a function of ATP with different amounts of ADP present. (A) 1 μM Rho-MatB T167A; (B) 1 μM Rho-MatB T303A; (C) 1 μM Rho-MatB S170A. Apart from the data labeled “no ADP”, the total nucleotide (ADP + ATP) for the weak-binding variants was kept constant at the micromolar concentrations shown. For Rho-MatB S170A, ADP was added at the concentration shown. Solution conditions were as in Fig 2. One representative data set is shown, normalized to the intensity in the absence of ATP. Linear fits to the data demonstrate approximate linear dependence over the range measured.

In assays in which ATP is being measured, ADP may also be present, at relatively high concentration, as ATP and ADP interconversion is part of many metabolic reactions. In order to show examples of how the presence of ADP affected the fluorescence signals of the variants to ATP, response curves were constructed at different levels of ADP (Fig 5). The total concentration of nucleotide (ATP + ADP) was held constant, to mimic partial interconversion of the two nucleotides. In the absence of ADP the response was approximately linear up to a concentration of one half the *K*_d_-value of ATP: of course, these are equivalent to the first part of the binding curves in Fig 2. For both the weak-binding variants there was still a good response with 500 μM ADP present, the highest concentration tested. At this high ADP, the response was ~50% of that without ADP. This is due in part to ADP binding itself and in part due to the lower response overall when high ADP is present, as reported previously for the parent protein [2]. As with all such probes, a calibration curve will be needed for the particular experimental conditions. However, the data in Fig 5 indicates that high concentrations of ADP can be accommodated and also indicates the ATP ranges that are readily measurable.

### Association kinetics of the weak-binding variants

Although a complete kinetic analysis was beyond the scope of this work, it was important to gauge how the main binding kinetics changed from the parent Rho-MatB and so relate that to potential response time with ATP. Association transients were obtained under pseudo-first order conditions by rapidly mixing each Rho-MatB variant with a large excess of ATP and following fluorescence with time (Fig 4 and S3 Fig). Qualitatively the weak-binding variants were similar to the parent [2], showing two distinct phases. The rate constants obtained are shown in Table 2.

The fast phase (Fig 4) had a rate constant that increased linearly with ATP concentration and was interpreted as binding. This association rate constant, determined from the gradient, varied very little between the parent and the different variants. The dissociation rate constant could be estimated from the intercept and was the main difference between variants.

The slow phase (S3 Fig) had a rate constant that did not change significantly with concentration: an average value is shown in Table 2. This phase was interpreted, based on the very similar data with the parent Rho-MatB, as a conformation change of the apoprotein prior to binding, as shown in the scheme in Fig 5C [2]. This suggested that the extra mutations did not affect the conformation change significantly.

Overall, the main effect of these two active site mutations is on the dissociation kinetics, whereby the affinity for ATP is modified.

### Properties of the tight-binding variant, S170A

In contrast to the two mutations on the phosphate binding loop, described above, the S170A variant, also on this loop, strengthens binding. This amino acid does not appear to interact with the triphosphate directly, unlike the hydrogen bonding between T167A and T303A and the γ- and α-phosphate of ATP, respectively. ATP and ADP binding was measured for (S170A)Rho-MatB, as described above for the other variants.

The changes to the fluorescence spectra (Fig 3C) and absorbance spectra (S2C Fig) were similar to those described above for the weak-binding variants and for the parent Rho-MatB [2]. The fluorescence change was slightly higher than the parent (Table 2). Titration with ATP gave a *K*_d_ of 3.8 μM, approximately two fold tighter than the parent (Fig 2C). However, because ADP binding was apparently weakened by this mutation compared to the parent, the selectivity for ATP over ADP was about 3-fold better. That can be seen in Fig 5C: at 100 μM ADP, ~100-fold higher than the ATP being measured, the fluorescence was only slightly reduced from that with ATP alone. This change in relative affinities is significant for potential use to measure ATP in assays where ADP is present at high ratio over ATP.

Like the two, weak-binding variants, the association kinetics were measured for the S170A Rho-MatB (Fig 4) to obtain the second order association rate constant (Table 2). This rate constant was very similar for all three variants and the parent Rho-MatB. The second phase, likely to represent a conformation change prior to ATP binding as described above, was also very similar to the other variants and parent Rho-MatB (S3 Fig and Table 2). The value of *k*_*-2*_ (noting the error) is consistent with this being a major factor in the change in dissociation constant relative to the parent. However, a change of only two-fold would be difficult to rationalize in more detail.

### Assessment of AMP binding

**Fig 6:**
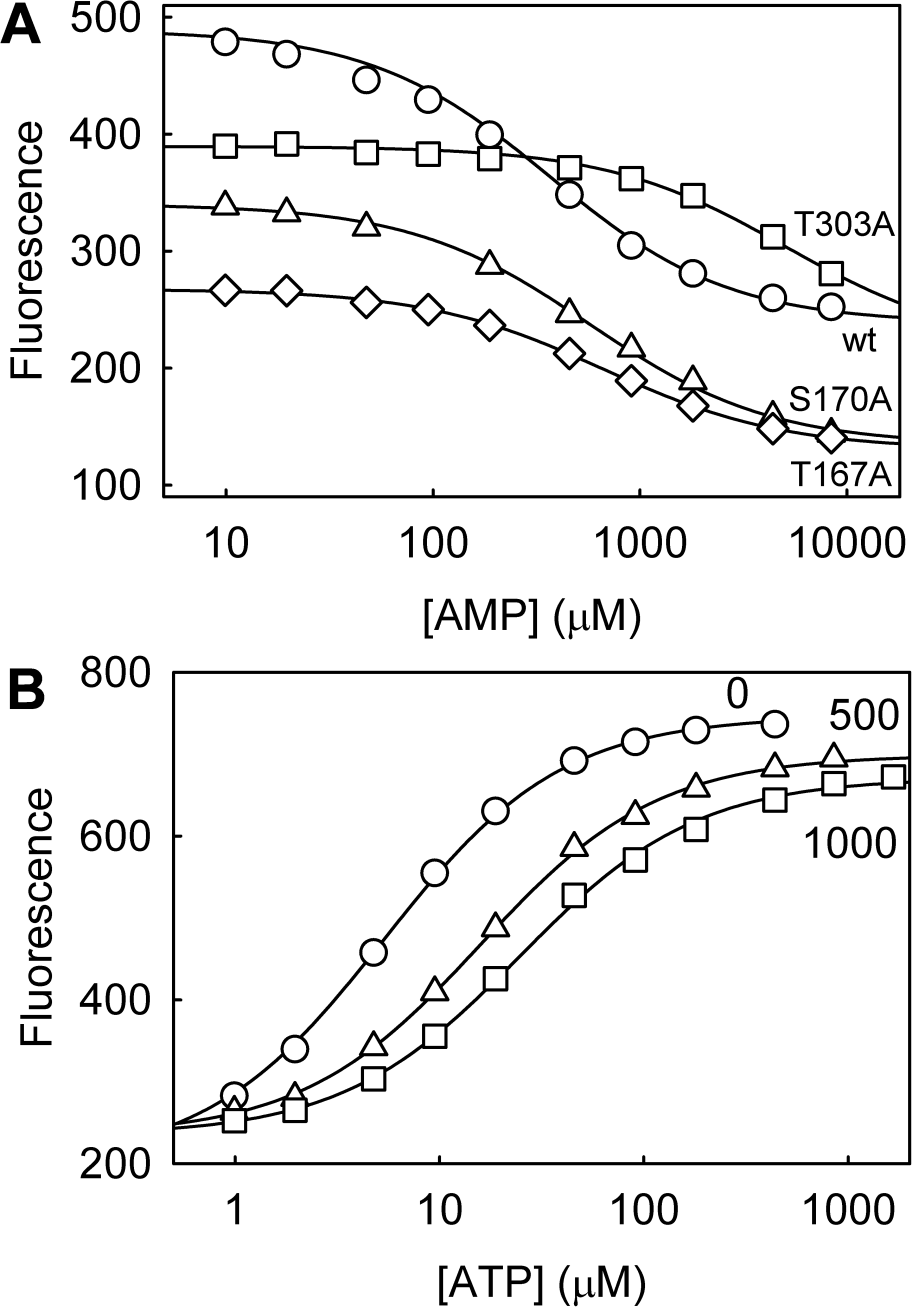
Competition titrations between ATP and AMP binding to Rho-MatB variants. Titrations were under conditions as Fig 2, using 0.5 μM of the Rho-MatB variant. (A) Titration of AMP into Rho-MatB complex with a fixed concentration of ATP: 7 μM for the parent Rho-MatB (“wt”) and S170A, 50 μM for T303A and 150 μM for T167A. One representative data set is shown. Fluorescence changes were fit to *A*_0_[ATP]/(*K*' +[ATP]), where the apparent dissociation constant for ATP, *K*' = *K*_ATP_(1 + [AMP]/*K*_AMP_). *K*_ATP_ and *K*_AMP_ are the actual dissociation constants for ATP and AMP and *A*_0_ is the total fluorescence change at that ATP concentration. Taking the previously obtained values for *K*_ATP_, the values of *K*_AMP_ were 169 ± 23 μM for the parent Rho-MatB, 225 ± 15 μM for S170A, and 300 ± 12 μM for T167A. T303A binding AMP was too weak to obtain a value. (B) Titrations at fixed AMP demonstrate there is little or no fluorescence change with AMP and titrating in ATP give close to the full fluorescence change. Data were fitted to the equation above, giving *K*_AMP_ as 233 ± 11 μM using 500 μM AMP and 255 ± 16 μM at 1000 μM AMP.

Although, as described above, ADP binds weakly, binding of natural ligands of RpMatB could, in principle, also affect its use as a biosensor for ATP, if such ligands are present in the assay solution. Previously, malonate and coenzyme A were shown not to give a fluorescence change with the parent Rho-MatB. Another potential ligand that may affect ATP binding is AMP and this showed a small fluorescence change on addition to the parent Rho-MatB [2]. Dissociation constants for AMP, both for the parent and each variant Rho-MatB, were determined by competition titrations (Fig 6). These can potentially be performed by keeping ATP concentration constant and varying AMP or vice versa. Firstly, titrations were done by adding AMP to Rho-MatB, bound with a fixed concentration of ATP close to its dissociation constant (Fig 6A). As AMP increases, it displaces bound ATP. The data gave dissociation constants of ~200 μM for the parent and S170A variant, both of which bind ATP tightly. T167A had a dissociation constant of 300 μM, while the affinity of T303A for AMP was too weak to measure accurately. This reflects the fact that T303A mutation removes an interaction with the α-phosphate, likely to be in the same position in the structure as where the AMP phosphate binds.

Doing the competition titration the other way round, fixing AMP, caused a shift in apparent dissociation constant of ATP. This was demonstrated using the parent Rho-MatB (Fig 6B): the apparent dissociation constant was increased to 15.7 ± 0.5 μM. Alternatively, fitting the data as described in Fig 6 gave values for the AMP dissociation constant that were similar to those in Fig 6A. The amplitude of the signal changes only little with AMP, but the range of ATP concentrations over the maximal signal change, is increased as AMP increases

Addition of 500 μM AMP also changed the kinetics of ATP binding (Fig 4B), reducing the association rate constant several-fold to 0.24 ± 0.01 μM^-1^s^-1^. A decrease would be expected due to competition with the AMP. The second, slow phase was little effected (0.65 ± 0.02 s^-1^).

### Test assay

**Fig 7:**
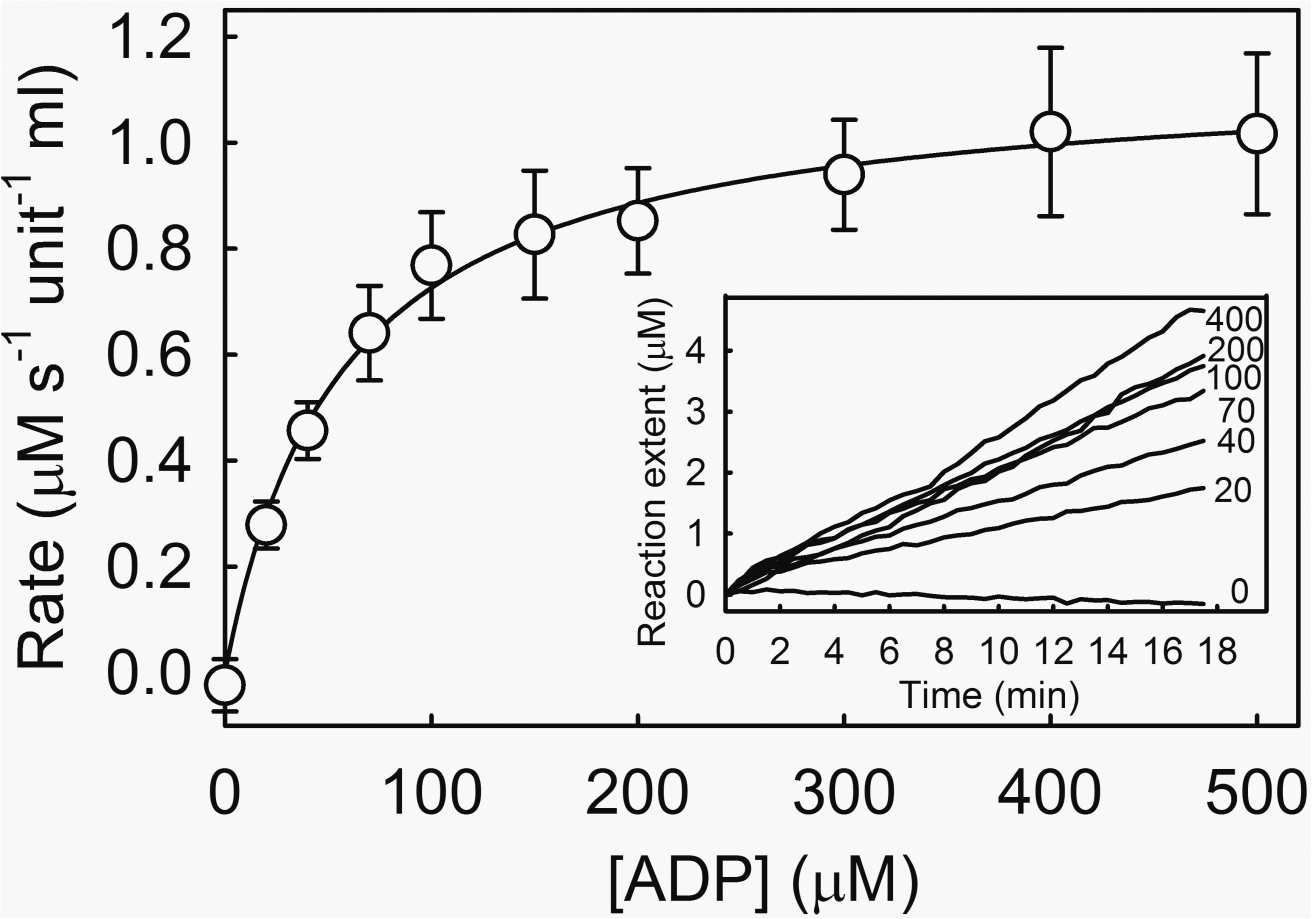
Test assay, measurement of the *K*_m_ of ADP with pyruvate kinase using T303A variant ATP biosensor. The assay was run in multiwell format in 50 mM HEPES buffer pH 7.2, 100 mM KCl, 10 mM MgCl_2_, 0.3 mg ml^-1^ bovine serum albumin at 25°C. The assay components were 500 μM phosphoenolpyruvate, 0.005 unit ml^-1^ pyruvate kinase, 1 μM T303A rho-MatB and the micromolar concentrations of ADP shown on the inset panel for an illustrative set of traces. The data are shown as ATP production rates, using calibrations done at 0–20 μM ATP at 0 μM ADP and with ADP to make 500 μM total nucleotide concentration. Intermediate calibrations were by interpolation. The average (with standard errors) are plotted for four independent measurements, giving *K*_m_ of 56 ± 4 μM.

We show one type of steady-state assay using a weak-binding variant, namely T303A, and a well understood enzyme system. This variant was used to measure the *K*_m_ for ADP with pyruvate kinase (Fig 7). This measurement would not have been possible with the original reagent, as it requires measurements with hundreds of micromolar ADP. The assay measured formation of ATP as a function of time and made use of calibrations of the fluorescence signal at 0 and 500 μM ADP.

## Conclusions

We have developed variants of Rho-MatB to change the range of ATP concentrations that can be measured, while maintaining a significant fluorescence change on ATP binding. Mutations in the ATP binding site gave us insights into the contributions of specific residues to ATP binding. The fluorescence labeling provided a signal to measure the effect of the amino acid side chain on the affinity of a protein-ligand complex. In particular, the effect of three mutations in the phosphate binding loop were studied in detail, as these gave desirable modifications to the Rho-MatB biosensor for measuring ATP. T167A and T303A decrease the affinity and so provide biosensors in the tens of micromolar ATP, up to >100 μM. These can be used substoichiometrically, for example at <1 μM, whereby the concentration of ATP is directly correlated to the fraction of the Rho-MatB in the high fluorescence, ATP-bound state.

In contrast, S170A increased ATP affinity, producing a more sensitive probe for ATP with a larger selectivity for ATP over ADP than the parent biosensor. The greater sensitivity comes from a combination of tighter binding and larger fluorescence enhancement.

Although care must be taken when using biosensors in the presence of potential ligands other than the analyte, the data with AMP suggest that this in itself does not prevent ATP measurements using RpMatB.

The additional variants present new opportunities for assay development, particularly real-time assays of ATP formation or depletion over a wide range of concentrations, so sensitivity can be optimized by choosing variants of Rho-MatB with particular dissociation constants. They provide additional resources to widen application over probes, for example, for ADP and P_i_, which products are likely to remain the choice analytes for straightforward kinase and ATPase assays. The types of reagentless biosensors, presented here, have a good combination of sensitivity and, in particular, selectivity, in this case particularly relative to ADP. As such they compare well with other types of biosensor, such as aptamer-based [26].

## Supporting information

S1 Fig Core motifs sequences. Sequence logos were created for ANL superfamily proteins, using WebLogo 3.4 (http://weblogo.threeplusone.com/). See Materials and Methods for details of sequence alignment. The proteins are listed below^1^ with their UniProt entry. Sequence conservation is indicated as the total height of each stack (measured in bits), while the relative height of bases in a stack reflects base frequencies at that position. The numbers correspond to the alignment position. The colour scheme is based on hydrophobicity: R, K, D, E, N, Q are blue; S, G, H, T, A, P are green; Y, V, M, C, L, F, I, W are black. The motifs shown are ones in which mutations were prepared: the sequence of RpMatB is also shown for each.
(PDF)

S2 Fig Absorbance spectra of variants of Rho-MatB with and without ATP. (A) 1 μM Rho-MatB T167A with and without 5 mM ATP; (B) 1 μM Rho-MatB T303A with and without 3 mM ATP; (C) 1 μM Rho-MatB S170A with and without 0.5 mM ATP. These ATP concentrations were saturating for the variant. Solutions were in 50 mM Hepes pH 7.0, 100 mM NaCl, 10 mM MgCl_2_, 0.3 mg ml^-1^ bovine serum albumin at 20°C.
(PDF)

S3 Fig Association kinetics of variants of Rho-MatB with excess ATP. Example time courses were obtained as in Fig 4 at various ATP concentrations, shown in micromolar for Rho-MatB T167A, T303A and S170A variants. While Fig 4 shows the fast phases of each time course, the equivalent slow phase are shown here. These were fit to single exponentials, whose rate constants varied little with ATP concentration. The average rate constants for this phase, measuring a conformation change as described in the main text, are in Table 2.
(PDF)
