# Supplementary material for: Development of a range of fluorescent reagentless biosensors for ATP, based on malonyl-coenzyme A synthetase
Source: PLoS One. 2017 Jun 21;12(6):e0179547. doi: 10.1371/journal.pone.0179547 (PMC5479551; doi:10.1371/journal.pone.0179547)
Supplement: S1 Fig — Sequence logos were created for ANL superfamily proteins, using WebLogo 3.4 (http://weblogo.threeplusone.com/). See Materials and Methods for details of sequence alignment. The proteins are listed below1 with their UniProt entry. Sequence conservation is indicated as the total height of each stack (measured in bits), while the relative height of bases in a stack reflects base frequencies at that position. The numbers correspond to the alignment position. The colour scheme is based on hydrophobicity: R, K, D, E, N, Q are blue; S, G, H, T, A, P are green; Y, V, M, C, L, F, I, W are black. The motifs shown are ones in which mutations were prepared: the sequence of RpMatB is also shown for each. (PDF) [file pone.0179547.s001.pdf]

S1 Fig. Core motifs sequences

Sequence logos were created for ANL superfamily proteins, using WebLogo 3.4 (http://weblogo.threeplusone.com/). See Materials and Methods for details of sequence alignment. The proteins are listed below[1] with their UniProt entry. Sequence conservation is indicated as the total height of each stack (measured in bits), while the relative height of bases in a stack reflects base frequencies at that position. The numbers correspond to the alignment position. The colour scheme is based on hydrophobicity: R, K, D, E, N, Q are blue; S, G, H, T, A, P are green; Y, V, M, C, L, F, I, W are black. The motifs shown are ones in which mutations were prepared: the sequence of RpMatB is also shown for each.

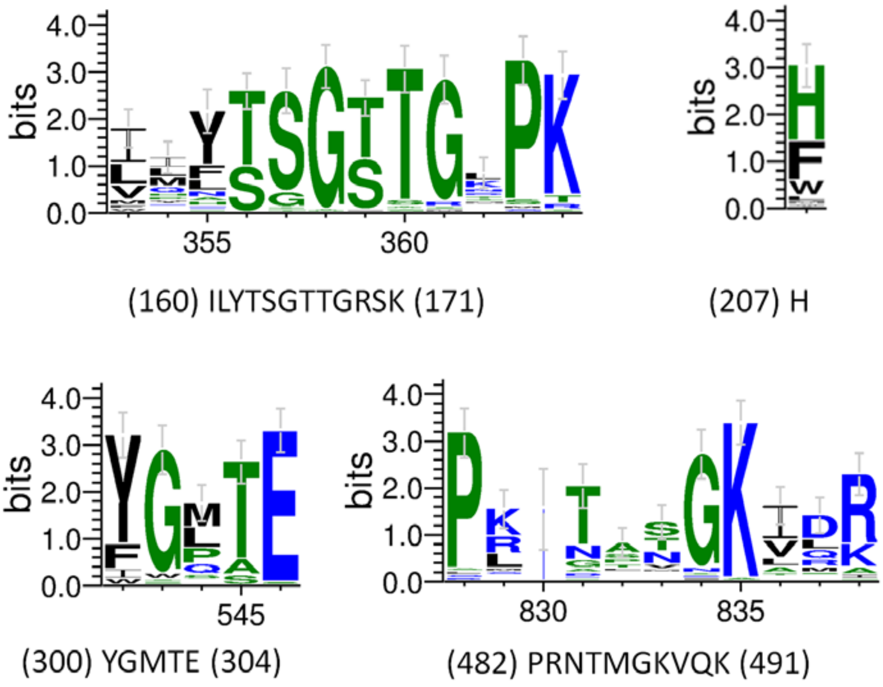

[1]Proteins aligned were*:* *P. pyralis* Luciferin 4-monooxygenase (P08659) - **PpLuc**; *B. brevis* Gramicidin S synthase I (P0C062) - **BbPheA**; *B. subtilis* 2,3-dihydroxybenzoate-AMP ligase (P40871, residues 3 to 556) - **BsDhbE**; *S. enterica* Acetyl-coenzyme A synthetase (Q8ZKF6) - **SeAcs**; *S. cerevisiae* Acetyl-coenzyme A synthetase 1 (Q01574) - **ScAcs**; *A. sp AL3007* 4-chlorobenzoyl CoA ligase (Q8GN86) - **AsCBL**; *T. thermophilus* Long-chain-fatty-acid-CoA ligase (Q5SKN9) - **TtLCFAcs**; *L. cruciata* Luciferin 4-monooxygenase (P13129) - **LcLuc**; *B. xenovorans* Benzoate-coenzyme A ligase (Q13WK3) - **BxBCL**; *H. sapiens* Acyl-coenzyme A synthetase ACSM2A (Q08AH3) - **HsACSM2A**; *B. subtilis* Surfactin synthase subunit 3 (Q08787, residues 465 to 956) - **BsArfA-C**; *B. cereus* D-alanine-poly(phosphoribitol) ligase subunit 1 (Q81G39) - **BcDltA**; *B. subtilis* D-alanine-poly(phosphoribitol) ligase subunit 1 (P39581) - **BsDltA**; *M. acetivorans* AMP-binding protein (Q8TLW1) - **MaAAE**; *M. tuberculosis* Long-chain-fatty-acid-AMP ligase FadD28 (P9WQ59) - **MtFAAL28**; *A. fulgidus* Long-chain-fatty-acid-CoA ligase (O30147) - **AfFadD1**; *B. cenocepacia* Phenylacetate-coenzyme A ligase (B4E7B5) - **BcPAAK1**; *B. cenocepacia* Phenylacetate-coenzyme A ligase (B4EL89) - **BcPAAK2**; *P. tomentosa* 4-coumarate:CoA ligase (Q941M3) - **Pt4CL**; *S aureus* 2-succinylbenzoate-CoA ligase (P63526) - **SaOSBC**; *E. coli* 2-succinylbenzoate-CoA ligase (P37353) - **EcMenE**; *B. subtilis* 2-succinylbenzoate-CoA ligase (P23971) - **BsMenE**; *E. festucae* Epichloenin A synthetase (K7NCP5, residues 618 to 1174) - **EfSidN**; *R. palustris* Putative long-chain-fatty-acid CoA ligase (Q6NAE3) - **RpFAAL**; *E. coli* Putative saframycin Mx1 synthetase B (A0A0H2VDD9) - **EcFAAL**; *L. pneumophila* Saframycin Mx1 synthetase B (Q5ZTD3) - **LpFAAL**; *S. pyogenes* D-alanine-poly(phosphoribitol) ligase subunit 1 (Q5XBN5) - **SpDAE**; *P. agglomerans* EhpF (Q8GPH0) - **PaEhpf**; *S. coelicolor* Putative fatty acid synthase (Q9L0A2) - **ScMatB**; *A. baumannii* BasE (no UniProt ID available, used PDB sequence) - **AbBasE**; *A. thaliana* 4-coumarate-CoA ligase 1 (Q42524) - **At4CL1**; *M. tuberculosis* Long-chain-fatty-acid-CoA ligase FadD13 (P9WQ37) - **MtFAAL13**; *L. turkestanicus* Luciferase (Q5UFR2) - **LtLuc**; *E. coli* Enterobactin synthase component E (P10378) - **EcEntE**; *P. aearuginosa* Uncharacterized protein (Q9I4B7) - **Pa1221**; *R. palustris* Malonyl CoA synthetase (Q6ND88) - **RpMatB**; *B. thetaiotaomicron* Phenylacetate-coenzyme A ligase (Q8AAN6) - **BtPCL**; *Streptomyces sp.* NRPS adenylation protein CytC1 (no UniProt ID available, used PDB sequence) - **SpCytC1**; *M. tuberculosis* Fatty-acid-CoA ligase FadD10 (A0A0J9WZY3) - **MtFadD10**; *C. scindens* Bile acid-coenzyme A ligase (P19409) - **CsBaiB**; *R. palustris* Benzoate-coenzyme A ligase (Q93TK0) - **RpBCL**; *D. fermentans* Acyl-CoA synthetase (AMP-forming)/AMP-acid ligase II-like protein (C6W5A4) - **DfACS**; *Streptomyces halstedtii* Non-ribosomal peptide synthetase (Q76KY2) - **ShVinN**; *P. agardhii* ApnAA1 (G0WVH3) - **PaApnA**; *V. cholera* Enterobactin synthetase component F-related protein (Q9KRQ7) - **VcAlmE**; *M. aeruginosa* McyG protein (A8YJW1, residues 1 to 643) - **MaMcyG**; *S. aurantiaca* Anthranilate-CoA ligase (F3Y661) - **SaAuaeEII**.
