# Supplementary material for: Development of a range of fluorescent reagentless biosensors for ATP, based on malonyl-coenzyme A synthetase
Source: PLoS One. 2017 Jun 21;12(6):e0179547. doi: 10.1371/journal.pone.0179547 (PMC5479551; doi:10.1371/journal.pone.0179547)

S2 Fig. Absorbance spectra of variants of Rho-MatB with and without ATP

(A) 1 µM Rho-MatB T167A with and without 5 mM ATP; (B) 1 µM Rho-MatB T303A with and without 3 mM ATP; (C) 1 µM Rho-MatB S170A with and without 0.5 mM ATP. These ATP concentrations were saturating for the variant. Solutions were in 50 mM Hepes pH 7.0, 100 mM NaCl, 10 mM $MgCl_2$, 0.3 mg ml^{-1} bovine serum albumin at 20 °C.

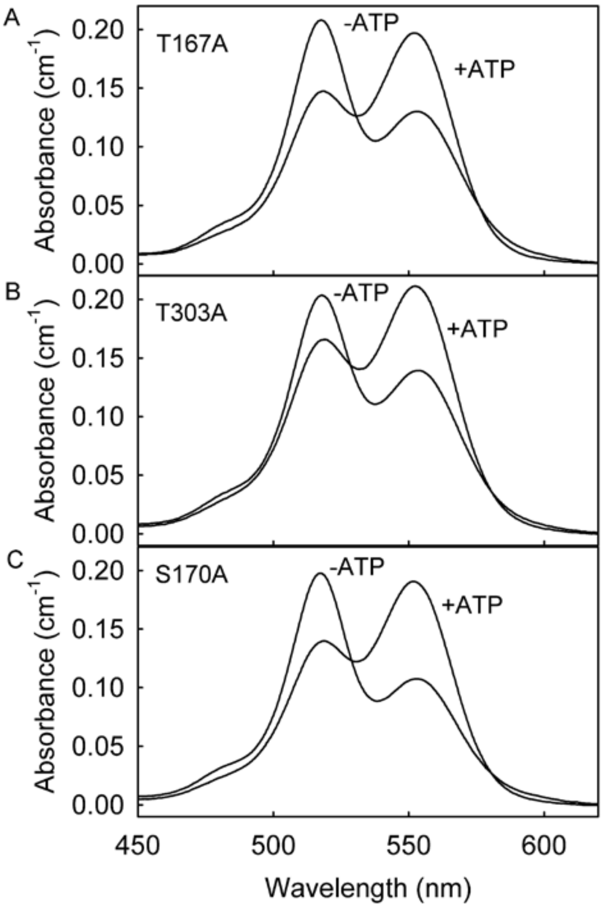

Supplement: S2 Fig — (A) 1 μM Rho-MatB T167A with and without 5 mM ATP; (B) 1 μM Rho-MatB T303A with and without 3 mM ATP; (C) 1 μM Rho-MatB S170A with and without 0.5 mM ATP. These ATP concentrations were saturating for the variant. Solutions were in 50 mM Hepes pH 7.0, 100 mM NaCl, 10 mM MgCl2, 0.3 mg ml-1 bovine serum albumin at 20°C. (PDF) [file pone.0179547.s002.pdf]
