# Supplementary material for: Development of a range of fluorescent reagentless biosensors for ATP, based on malonyl-coenzyme A synthetase
Source: PLoS One. 2017 Jun 21;12(6):e0179547. doi: 10.1371/journal.pone.0179547 (PMC5479551; doi:10.1371/journal.pone.0179547)

S3 Fig. Association kinetics of variants of Rho-MatB with excess ATP

Example time courses were obtained as in Fig 4 at various ATP concentrations, shown in micromolar for Rho-MatB T167A, T303A and S170A variants. While Fig 4 shows the fast phases of each time course, the equivalent slow phase are shown here. These were fit to single exponentials, whose rate constants varied little with ATP concentration. The average rate constants for this phase, measuring a conformation change as described in the main text, are in Table 2.

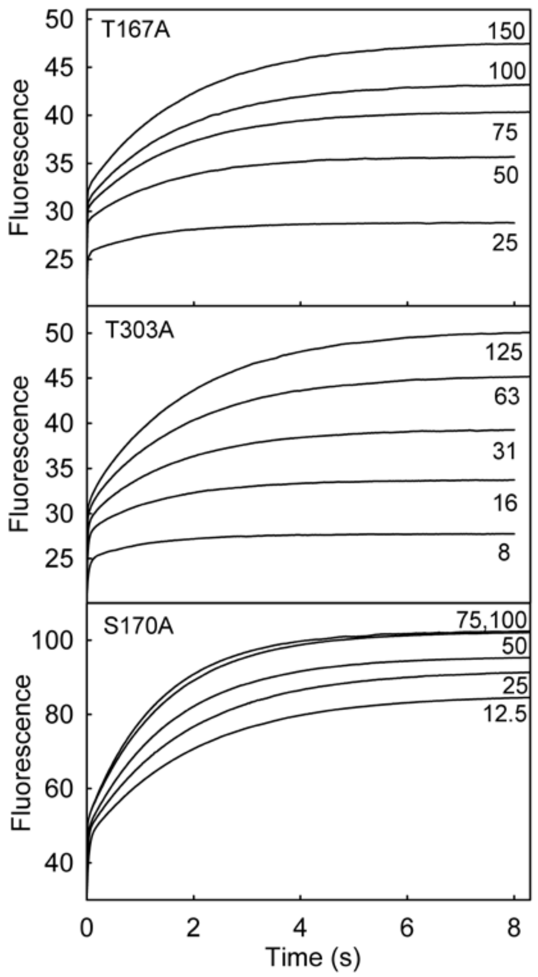

Supplement: S3 Fig — Example time courses were obtained as in Fig 4 at various ATP concentrations, shown in micromolar for Rho-MatB T167A, T303A and S170A variants. While Fig 4 shows the fast phases of each time course, the equivalent slow phase are shown here. These were fit to single exponentials, whose rate constants varied little with ATP concentration. The average rate constants for this phase, measuring a conformation change as described in the main text, are in Table 2. (PDF) [file pone.0179547.s003.pdf]
